# Supplementary material for: Label-free intratissue activity imaging of alveolar organoids with dynamic optical coherence tomography
Source: Biomed Opt Express. 2023 Apr 27;14(5):2333–51. doi: 10.1364/BOE.488097 (PMC10191660; doi:10.1364/BOE.488097)
Supplement: Supplementary file 1 [file boe-14-5-2333-s001.pdf]

Supplemental Document

Biomedical Optics EXPRESS

Label-free intratissue activity imaging of alveolar organoids with dynamic optical coherence tomography: supplement

RION MORISHITA,[1] TOSHIO SUZUKI,[2,3] PRADIPTA MUKHERJEE,[1] IBRAHIM ABD EL-SADEK,[1,4] YIHENG LIM,[1] ANTONIA LICHTENEGGER,[1,5] SHUICHI MAKITA,[1] KIRIKO TOMITA,[1] YUKI YAMAMOTO,[3] TETSUHARU NAGAMOTO,[3] AND YOSHIAKI YASUNO[1,*]

[1] *Computational Optics Group, University of Tsukuba, Tsukuba, Ibaraki 305-8573, Japan*
[2] *Department of Medical Oncology, Faculty of Medicine, University of Tsukuba, Ibaraki 305-8575, Japan*
[3] *HiLung Inc., Kyoto, Japan*
[4] *Department of Physics, Faculty of Science, Damietta University, New Damietta City 34517, Damietta, Egypt*
[5] *Center for Medical Physics and Biomedical Engineering, Medical University of Vienna, Währinger Gürtel 18-20, 4L, 1090, Vienna, Austria*
https://optics.bk.tsukuba.ac.jp/COG/
[*] *yoshiaki.yasuno@cog-labs.org*

This supplement published with Optica Publishing Group on 27 April 2023 by The Authors under the terms of the Creative Commons Attribution 4.0 License in the format provided by the authors and unedited. Further distribution of this work must maintain attribution to the author(s) and the published article's title, journal citation, and DOI.

Supplement DOI: https://doi.org/10.6084/m9.figshare.22664452

Parent Article DOI: https://doi.org/10.1364/BOE.488097

Label-free intratissue activity imaging of alveolar organoids with dynamic optical coherence tomography: supplemental document

In this supplementary material, we provide eleven figures, Figs. S1–S11. These figures show the LIV images of the alveolar organoids at several depth, and supplement Fig. 3 of the full-length manuscript. These samples are normal and bleomycin-model alveolar organoids at Days +3, +7, +10, +16, +17,and +18, while Fig. 3 of the full-length manuscript presents the normal organoid at Day +3. The field of view of the *en face* images (square panes) is 3 mm $\times$ 3 mm. The depth-independent distribution of the alveoli and fibroblasts were found in all samples.

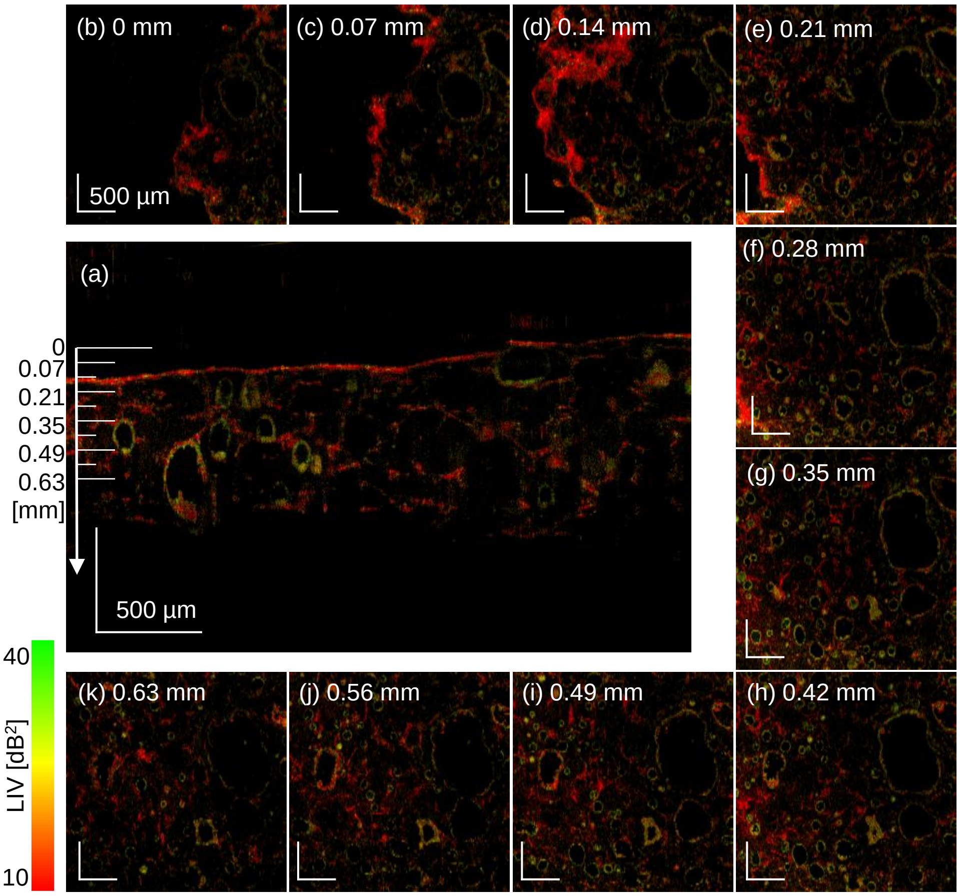

Fig. S1. The LIV *en face* images of Day +3 bleomycin-model organoid at several depths (square panes, FOV is 3 mm × 3 mm). The images were extracted at the depth of every 0.07 mm, and the depth positions are indicated in the cross-sectional LIV image at the center. The alveoli with high-LIV (green or green-red mixture) borders, which are possibly alveolar epithelium, and the fibroblasts are uniformly distributed in 3D.

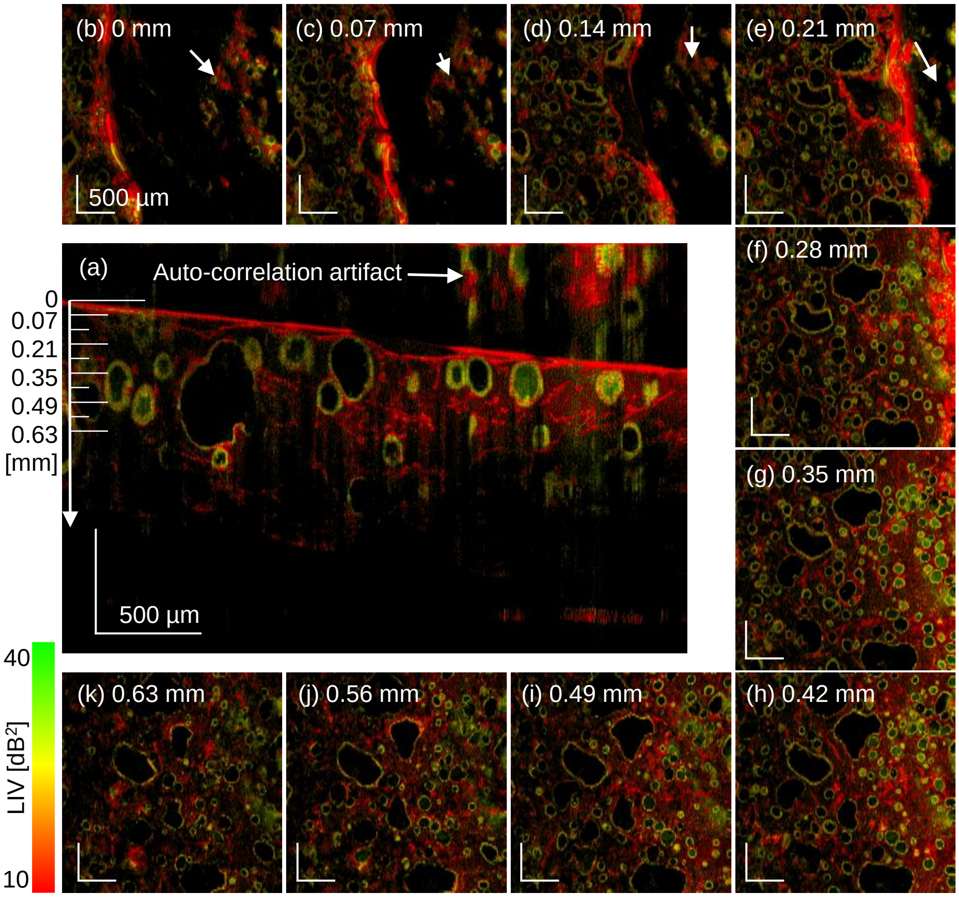

Fig. S2. The case of Day +7 normal organoid. The appearances indicated by white arrows are auto-correlation artifacts. Here after, all figures are presented in the same manner with Fig. S1.

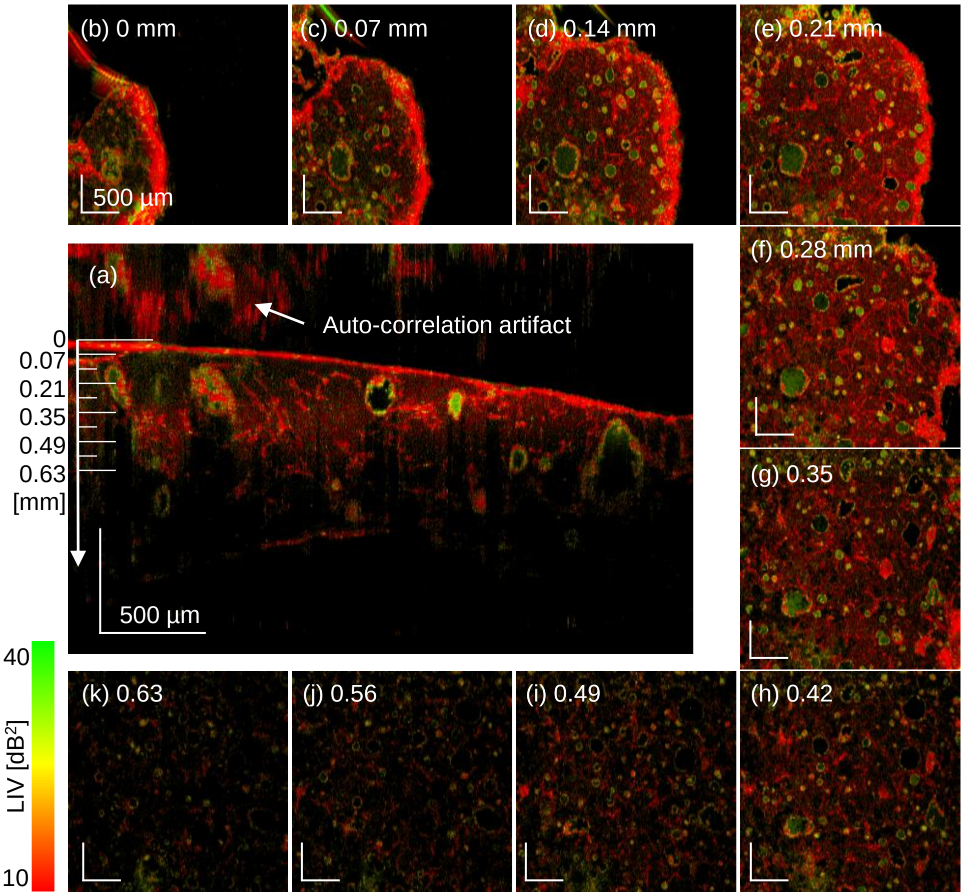

Fig. S3. The case of Day +7 bleomycin-model organoid.

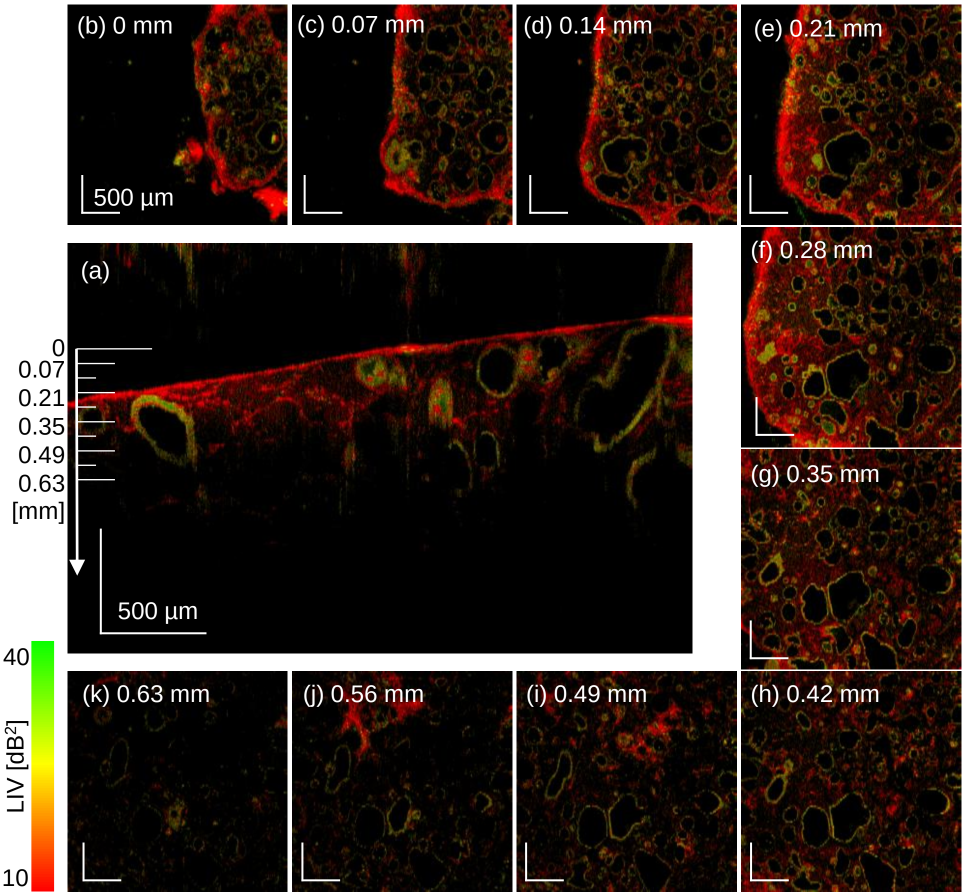

Fig. S4. The case of Day +10 normal organoid.

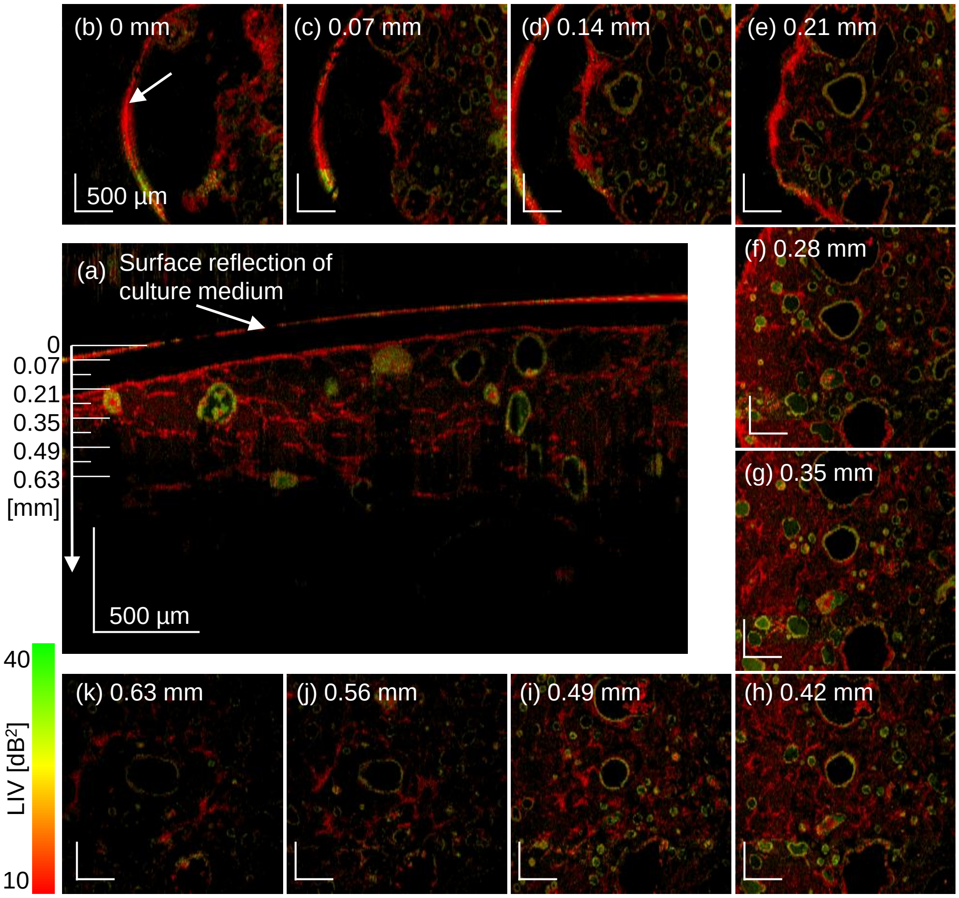

Fig. S5. The case of Day +10 bleomycin-model organoid. The appearances indicated by white arrows are surface reflection of culture medium.

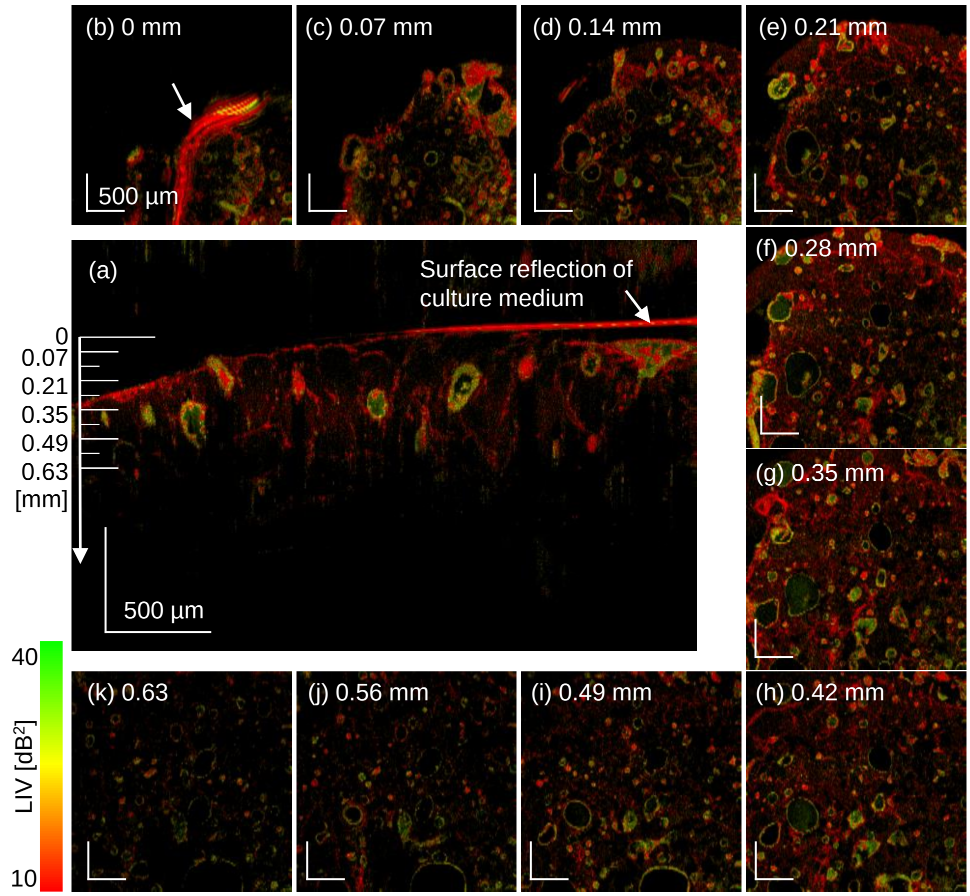

Fig. S6. The case of Day +16 normal organoid. The appearances indicated by white arrows are surface reflection of culture medium.

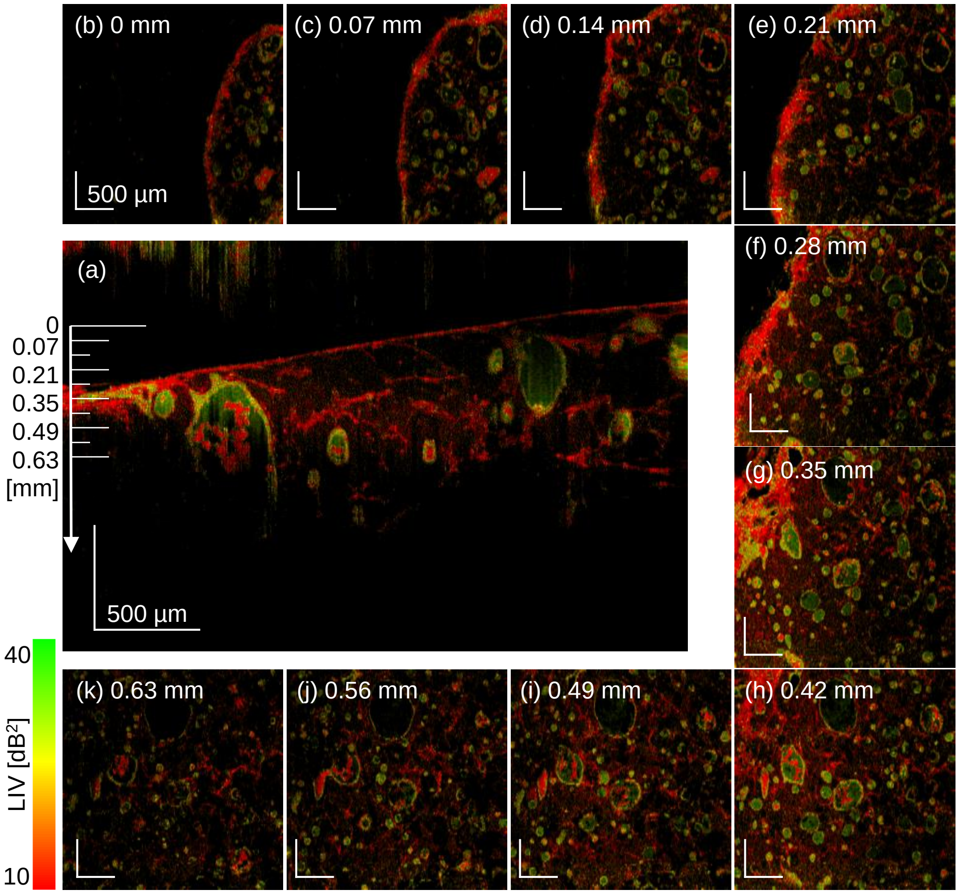

Fig. S7. The case of Day +16 bleomycin-model organoid.

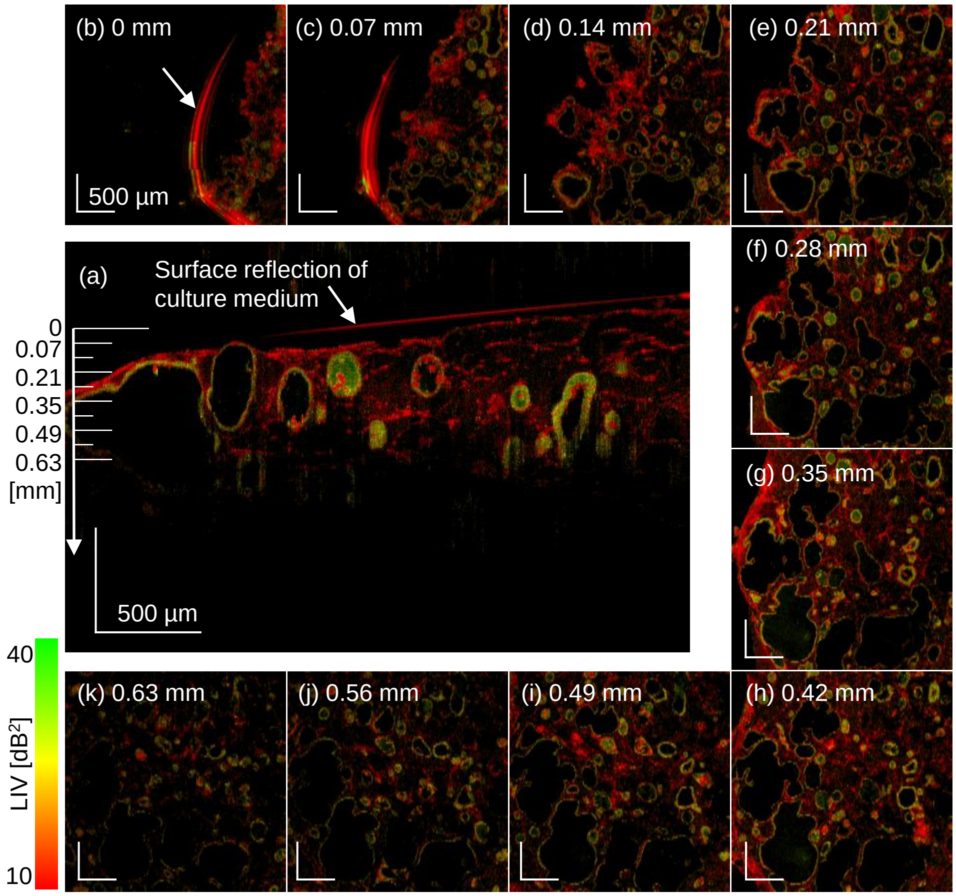

Fig. S8. The case of Day +17 normal organoid. The appearances indicated by white arrows are surface reflection of culture medium.

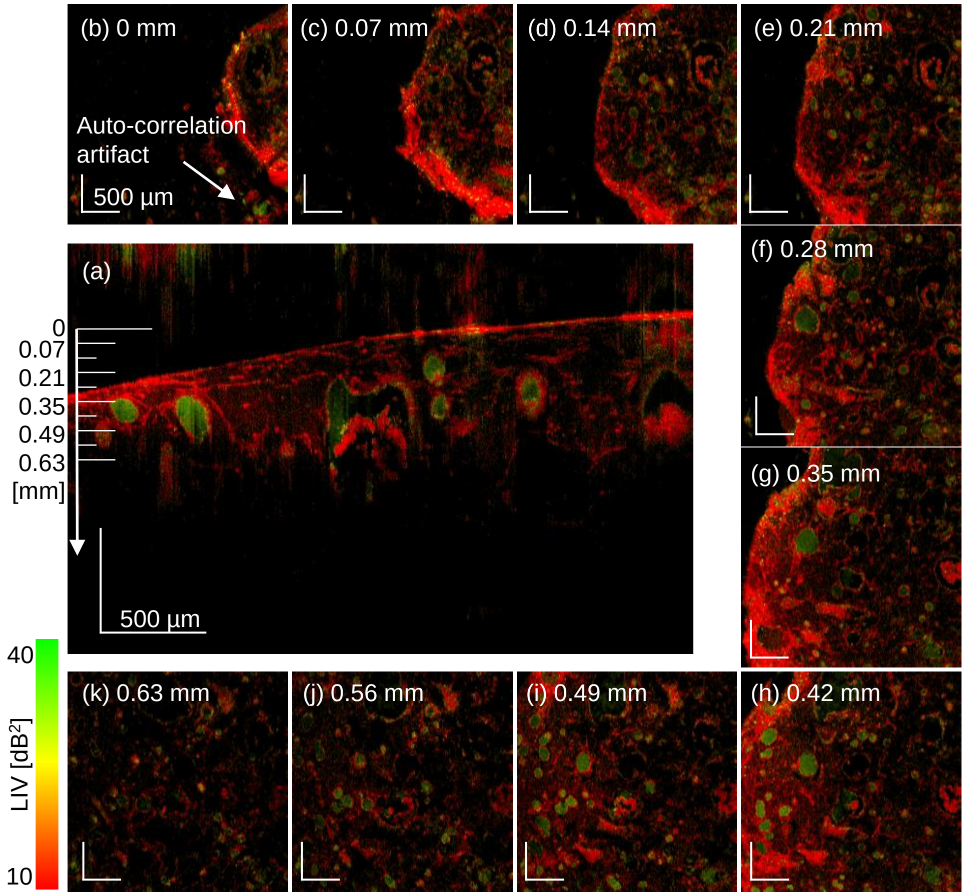

Fig. S9. The case of Day +17 bleomycin-model organoid. The appearance indicated by a white arrow is an auto-correlation artifact.

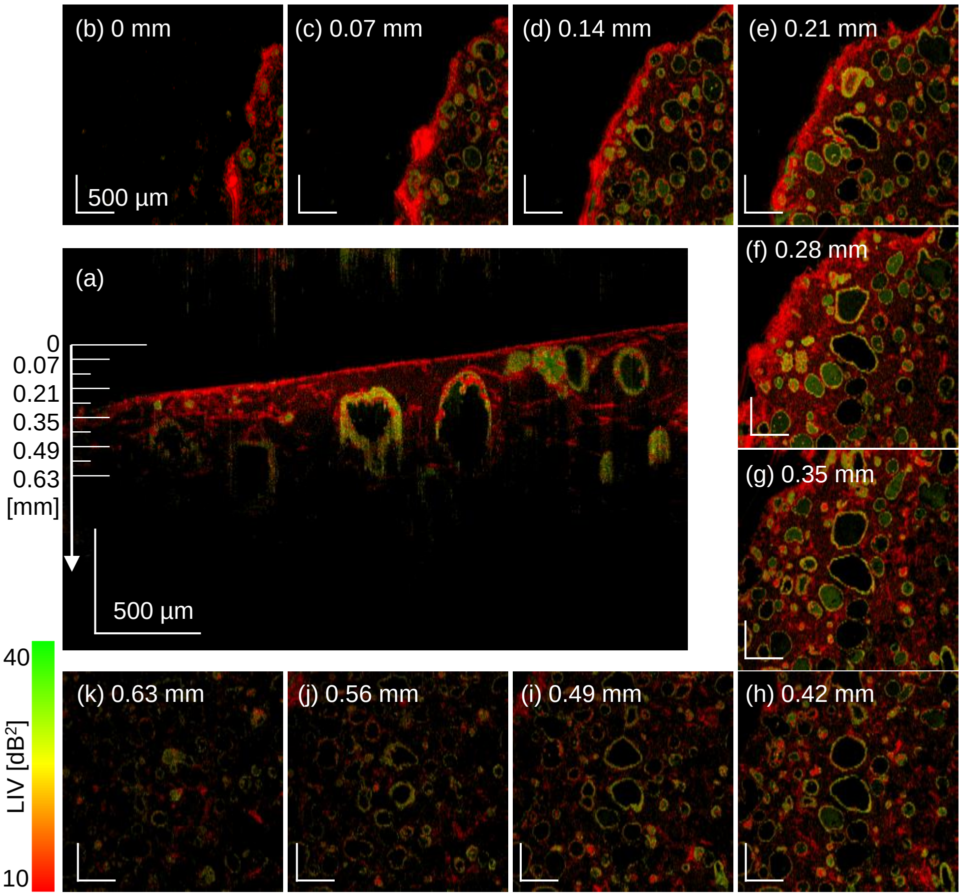

Fig. S10. The case of Day +18 normal organoid.

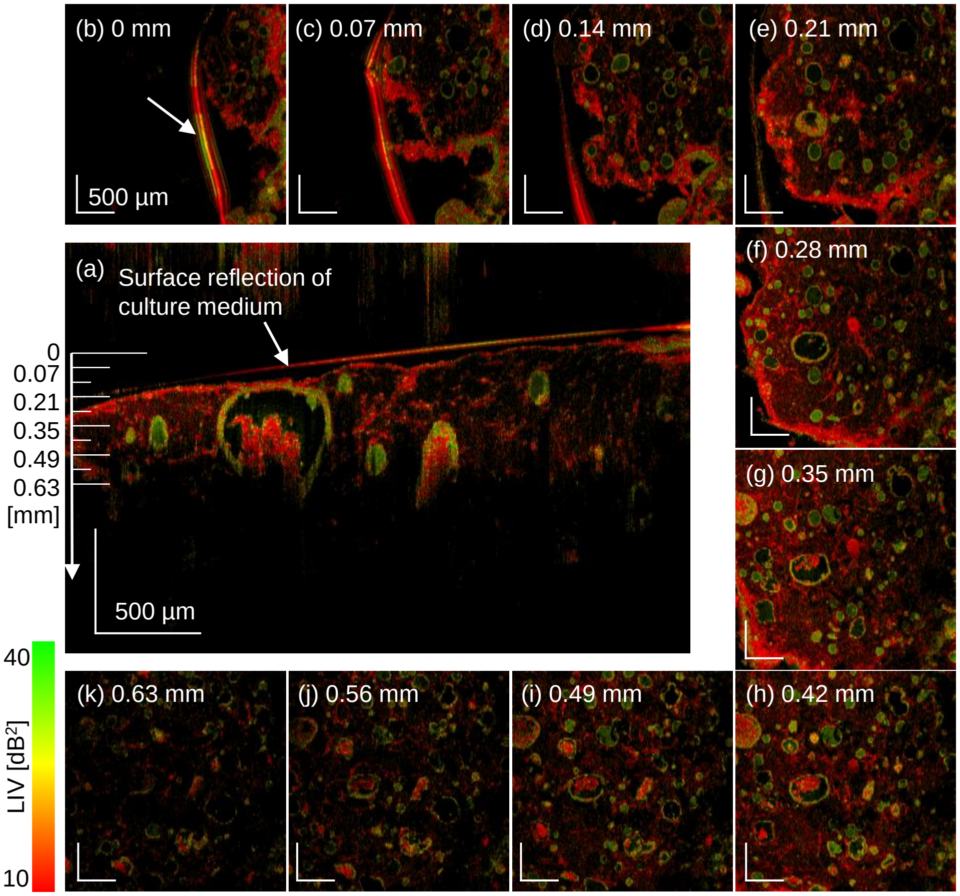

Fig. S11. The case of Day +18 bleomycin-model organoid. The appearances indicated by white arrows are surface reflection of culture medium.
